# Editorial: Minimally-invasive treatment for genitourinary cancers: what comes next?

**DOI:** 10.3389/fonc.2023.1285730

**Published:** 2023-09-19

**Authors:** Francesco Chierigo, Rocco Simone Flammia, Guglielmo Mantica

**Affiliations:** ^1^ Department of Urology, IRCCS Ospedale Policlinico San Martino, Genova, Italy; ^2^ Department of Surgical and Diagnostic Integrated Sciences (DISC), University of Genova, Genova, Italy; ^3^ Department of Maternal-Child and Urological Sciences, Sapienza Rome University, Policlinico Umberto I Hospital, Rome, Italy

**Keywords:** minimally invasive & robotic surgery, genitourinary cancer (GU cancer), bladder cancer (BCa), prostate cancer (PCa), kidney cancer

Due to its heterogeneous nature, urology has been one of the branches most affected by technological evolution in the surgical field, in particular with the introduction of laparoscopy, robotic surgery, ever-smaller endoscopes, and precise lasers. Over the years, this has affected the treatment of not only benign urological diseases but, obviously, also genitourinary cancers. In our age, the goal of surgical treatment is not only to cure, if possible, genitourinary tumors, but also to carry out this treatment using a method that is as minimally invasive as possible. In this special series, we will deal, mainly with some reviews and original research, with some of the technological advances currently available and in progress in the minimally invasive management of genitourinary tumors.

Three contributions are focused on the treatment of renal neoplasms. In particular, the original article published by Bianchi et al. explores the importance of using 3D models for the preoperative planning of robot-assisted partial nephrectomies, with a particular focus on improving outcomes. Similarly, the systematic review proposed by Chen et al. focuses on the minimally invasive surgical treatment of special types of renal tumors such as hilar, endophytic, and cystic. Wang et al. proposed an evidence-based analysis aimed at comparing the functional and oncological outcomes of patients with complex renal tumors undergoing two different minimally invasive approaches: robotic surgery and pure laparoscopic surgery. The authors concluded that in patients with complex renal tumors (RENAL score ≥7), robotic partial nephrectomy is superior to laparoscopic partial nephrectomy in decreasing the operative time, warm ischemia time, length of stay, transfusion rate, change in eGFR, and the incidence of intraoperative complications while maintaining oncological control and avoiding a decline in renal function.

Two studies are focused on upper tract urothelial carcinoma (UTUC). Lindner et al. proposed a retrospective study evaluating the follow-up protocols after kidney-sparing surgery and nephroterectomy, which is still a matter of debate. Wu et al. proposed an interesting step-by-step procedure for total intracorporeal laparoscopic kidney autotransplantation in a patient with distal high-risk upper tract urothelial carcinoma.

Teoh et al. conducted a proof-of-concept study on endoscopic ultrasound (EUS)-guided biopsy of detrusor muscle in porcine bladders. They used five porcine bladders in this experiment and were able to find detrusor muscle in 30 out of 37 biopsies (81.1%) without any bladder perforation. They concluded that an EUS-guided biopsy of the detrusor muscle could be performed during the initial cystoscopy session.

Lastly, Eubank et al. conducted a study with the aim of developing a safe and precise method for intraprostatic injection. The study was carried out on different prostate models. The authors found that intraprostatic injection using a porous needle allows for effective and predictable tissue distribution of the injectate in the prostate.

We would like to thank the editorial office, authors, reviewers, and all the readers for their efforts in putting together this series, hoping that it will be appreciated and useful by readers.

## Author contributions

FC: Writing – original draft, Writing – review & editing. RF: Writing – original draft, Writing – review & editing. GM: Writing – original draft, Writing – review & editing.

